# What Do Physicians Believe About the Way Decisions Are Made? A Pilot Study on Metacognitive Knowledge in the Medical Context

**DOI:** 10.5964/ejop.v11i4.979

**Published:** 2015-11-27

**Authors:** Paola Iannello, Valeria Perucca, Silvia Riva, Alessandro Antonietti, Gabriella Pravettoni

**Affiliations:** a Department of Psychology, Catholic University of the Sacred Heart, Milan, Italy; b Department of Health Sciences, University of Milan, Milan, Italy; c Interdisciplinary Centre for Research and Intervention on Decision (IRIDe Centre), Milan, Italy; d Institute of Oncology (IEO), Milan, Italy; University of South Wales, Newport, United Kingdom

**Keywords:** medical decision making, metacognition, self-awareness, emergency care, surgery, internal medicine

## Abstract

Metacognition relative to medical decision making has been poorly investigated to date. However, beliefs about methods of decision making (metacognition) play a fundamental role in determining the efficiency of the decision itself. In the present study, we investigated a set of beliefs that physicians develop in relation to the modes of making decisions in a professional environment. The Solomon Questionnaire, designed to assess metacognitive knowledge about behaviors and mental processes involved in decision making, was administered to a sample of 18 emergency physicians, 18 surgeons, and 18 internists. Significant differences in metacognitive knowledge emerged among these three medical areas. Physicians’ self-reports about the decision process mirrored the peculiarities of the context in which they operate. Their metacognitive knowledge demonstrated a reflective attitude that is an effective tool during the decision making process.

## Introduction

In the field of the psychology of decision making, judgments and choices are usually investigated in laboratory situations, making it difficult to uncover the actual development of diagnostic processes in emergency departments (ERs). In real work situations, it is necessary to consider the inherent limitations of our cognitive structure, making it impossible to examine all aspects of the situation. We must also consider the environmental restrictions that arise from a context, which is itself problematic for analyzing the interactions of crucial and unavoidable variables that constantly confront the clinician. These factors include: 1) the risks which has to be taken and the associated uncertainty when these risks are not known (Kahneman, Slovic, & Tversky, 1982); 2) the need to update information on the basis of experience; 3) the simplification of thinking strategies (heuristics) to speed assessment (Marewski & Gigerenzer, 2012); 4) stress and lack of time, which trigger the paradox of avoiding decision making or of concentrating on a single source of information (Allnutt, 2002); 5) an excess of confidence in one’s abilities and the consequent exclusion of other unpredicted intervening factors (Croskerry, 2002); and 6) high emotional impact, which can alter the calculation of probabilities and/or challenge rational selection processes (Hogarth, 1980).

Metacognition constitutes a possible framework for considering this multiplicity of critical factors in a coordinated manner (Yzerbyt, Lories, & Dardenne, 1998). Metacognition primarily concerns the knowledge that everyone has about his/her own mental processes. This knowledge encompasses the thinking strategies used to deal with a cognitive task (solving a problem, remembering a notion, etc.), the emotional states that accompany them, the perception of effort made by the individual, and the obstacles encountered. Metacognition also includes beliefs in personal attributes, one’s cognitive abilities, task characteristics (level of complexity, etc.), the context in which one works (time constraints, etc.), and the demands and expectations that others develop about an individual’s actions. Metacognition can also be linked to the self-criticism that goes with professional competence, vigilance about one’s performance, availing oneself of advice, managing organizational conflicts, and recalling failures that are transformed into caveats for future behavior. Metacognition refers to the ability to control one’s mental processes and the behaviors derived from them, based on a person’s awareness of such behaviors and on the conviction that he/she develops regarding his/her optimal method of proceeding.

In particular, metacognition related to decision-making processes – a little-studied aspect of clinical reasoning (Croskerry, 2000; Marcum, 2012; Pines, 2006) – refers to the level of knowledge an individual has regarding his/her method of making choices, the thinking strategies on which such choices are based, and the emotions experienced. Metacognition is the basis of the beliefs that individuals develop about the dynamics of the decision-making process, both with reference to their own personal characteristics (limits and strengths) and to the characteristics attributed to the ideal decision-maker. During the process of clinical decision making, metacognition seems to play a monitoring role, controlling or regulating the diagnostic/therapeutic decision (Marcum, 2012).

Recent research suggests that metacognition could be successfully utilized to correct “imbalances” that arise due to biases in clinical reasoning (Lucchiari & Pravettoni, 2012). Additionally, metacognition allows the physician to evaluate the clinical decision-making process and to determine whether the process is worth applying to future decisions (Marcum, 2012). A recent study suggests that individual differences in metacognitive competence may effectively predict the outcomes of clinical decision-making processes (Jackson & Kleitman, 2014). If the physician is fully aware of his/her method of decision making and reports adequate convictions about how decisions should be made, he/she should be able to exercise control over the decision-making process, plan it in a satisfactory manner, and change it when required.

### Aims

The objectives of this study were: 1) to explore the metacognitive knowledge that physicians possess about the way they make their decisions in the workplace; 2) to detect differences, if any, in metacognitive knowledge among various medical professions; and 3) to determine whether metacognitive knowledge differs according to the physician’s level of expertise.

## Methods

### Ethics Statements

Participants in the experiments described here were treated according to the ethical standards of the American Psychological Association.

In accordance with the procedure adopted in the investigators’ department, which seeks to avoid submitting projects to the Ethics Committee that cannot be problematic from the ethical point of view, this project was submitted to the Head of the Department to assess the need for submission to the Ethics Committee. Consultation with the Chairman of the Ethics Committee determined that the research did not require submission to the Ethics Committee.

The first page of the Solomon Questionnaire explicitly stated that participants would remain anonymous. Researchers had no way to identify the physicians, which would have been possible if written consent was obtained from participants. After explaining the purpose of the study, potential participants were asked whether everything was clear and whether they consented to participate.

### Participants

The questionnaire was administered to 54 physicians: 18 ER physicians, 18 general surgeons, and 18 internists. Physicians were located at four hospitals in Northern Italy: Ospedale di Borgosesia (Vercelli), Ospedale di Busto Arsizio (Varese), Ospedale San Carlo (Milano), and Ospedale Valduce (Como). The sample consisted of 36 men and 18 women, with more male surgeons and internists (83% men in surgery, 66% in internal medicine, and 50% in the ER).

Participant age ranged between 26 and 60 years (mean (*M*) = 44.92 years; standard deviation (*SD*) = 9.21 years). The mean ages of the three groups of specialists did not significantly differ (ER: *M* = 39.06 years, *SD* = 8.87 years; surgery: *M* = 46.51 years, *SD* = 9.65 years; internal medicine: *M* = 49.14 years, *SD* = 9.01 years; *F*(2, 53) = 1.67, *p* = .17, η^2^ = .02). Additionally, differences in age among the four hospitals were not significant (Borgosesia: *M* = 45.05 years, *SD* = 10.00 years; Busto Arsizio: *M* = 41.96 years, *SD* = 9.81 years; Milano: *M* = 46.62 years, *SD* = 9.81 years; Como: *M* = 43.32 years, *SD* = 9.63 years; *F*(3, 53) = 1.91, *p* = .20, η^2^ = .04).

The experience of the physicians within each specialty varied between 1 and 34 years and was significantly correlated with age (*r* = .91). Consequently, only data about experience were analyzed. Seniority was considered to reflect the level of expertise of the responders and was divided into three categories: low (< 9 years; *N* = 22), medium (9-23 years; *N* = 14), and high (> 23 years; *N* = 18). The three levels of expertise did not significantly differ among the four hospitals, χ^2^(6, *N* = 54) = 21.24.

Once we verified the homogeneity of the four subsamples in terms of physician age and level of expertise, the subsamples were pooled. Note that all four hospitals are situated within a 100-km radius, in an area with similar geographic and demographic features. The socioeconomic and educational levels of the patients in these hospitals are the same. All hospitals belong to the national health care system (none of them are private), and therefore they use the same rules and protocols.

### Materials

The Solomon Questionnaire (Colombo, Iannello, & Antonietti, 2010) was used to investigate metacognition in decision making. A version of the original questionnaire was adapted to the specific medical contexts of the present study.

The questionnaire (Appendix) consisted of two parts. In the first part, metacognitive knowledge about the personal strategy for making decisions was investigated on two levels. The descriptive-behavioral level (Items 1-6) defines the approach to decisions that the respondent generally applies during his/her working activity. The procedural-emotional level (Items 7-8) concerns the processes involved in decision making and the cognitive strategies and emotional reactions that are triggered during decision making. The second part of the questionnaire addressed the respondent’s metacognitive knowledge about the decision process in general, as well as the individual characteristics that, according to one’s own ideas, identify a “good decision-maker” (Items 9-15).

### Categorization of Responses to Open Questions

In order to analyze the responses to the open questions in the Solomon Questionnaire, responses were grouped into semantic categories (Table 1).

**Table 1:** Semantic Category System for Coding Questionnaire Responses

Items	Focus of the question	Corresponding question	Category
Item 2	“Typical” decision	2. Describe briefly three typical decisions of your working day	admittance/discharge therapy (prescribe or change) surgery or invasive tests diagnosis
Item 8a	Decision context	8a. Describe the general situation, i.e. the context in which you are required to make a decision	urgency routine management and organizational problems confusion (many patients at the same time, difficulty perceived)
Item 8b	First thought	8b. Which is your first thought?	not to cause harm focus on the patient (putting oneself in his/her shoes, concentrating attention on him/her) asking oneself questions/reflection
Item 8c	Personal feeling	8c. How do you feel when you make this decision?	calm/peaceful stressed/inadequate concentrated/absorbed other (rage/excitement/fear/powerless)
Item 8d	Which actions?	8d. What do you do to make this decision?	alternative investigation/consultation with others strategy (assessment of costs/benefits, preparation of an action plan) accumulated knowhow instinct
Item 8e	Confronting the problem with others?	8e. Do you face the situation by yourself or do you ask others for help/advice?	alone others it depends/if I do not have other means
Item 8f	Learning from the past?	8f. Do you basically employ solutions that turned out to be effective in the past or do you tend to try out new solutions?	effective in the past I experiment it depends both
Item 8g	Adhering to the first plan?	8g. Once you have made the decision, do you follow it or do you modify it (entirely or in part)? On the basis of which thoughts/reflections do you modify/don't modify your decision?	I adhere to the decision I change the decision during the process (due to new available data, changes in the condition of the patient)
Item 9	Characteristics of the “good decision-maker”	9. Which peculiarities characterize those people who are effective in taking their decisions?	experience/competence intuition/strength of character intelligence/metacognitive skills (equilibrium, reflection, self-awareness)
Item 10	Regret and the “good decision-maker”	10. A good decision maker is someone who never regrets his/her decision? Why?	it is possible to make mistakes it is possible to learn from one’s mistakes self-criticism (necessary)
Item 13	How to become a “good decision-maker”	13. How do you think a person can become a good “decision-maker”?	experience training/teachers increasing one’s own metacognitive awareness (understand how one makes decisions, awareness of one’s limits)
Item 14	The way to support “good decision making”	14. How can you help someone to make good decisions?	give advice develop self-esteem/metacognition act as an example
Item 15	Examples of a “good decision-maker”	15. Which could be a proper example of a “good decision-maker”? (you can mention historical or mythological characters, well-known people, but also colleagues, relatives or friends)	colleagues/superiors family members/friends current politicians historical figures fantasy/mythological figures

## Results

### Part 1 of the Questionnaire: Metacognitive Knowledge About the Personal Way of Making Decisions

Self-reported data from the overall sample (Table 2) indicate that a hospital physician makes an average of 31.5 decisions (range = 3-100 decisions) during a typical working day. A 6-h working day would yield ~5 decisions/hour. Typical decisions mainly concern diagnosis (37%) and therapy (28%). Of these decisions, 69.2% indicated that they involve direct physician responsibility, 56.3% are reversible, 38.9% said that they are mainly related to the physicians themselves, 23.4% require a lot of time to be reached, and 13% are accompanied by a feeling of regret – because the decision-maker believes, in retrospect, that a different choice would have been preferable. The context in which decisions was reported to be routine (37%) and urgent (31.5%). Above all, decisions seemed to be accompanied by an attempt to not cause harm and to avoid aggravating the patient's clinical situation. Feelings of stress and inadequacy or of peace and calm appeared to arise during the decision-making process. Of the responding physicians, 37% declared that they rely on their strategic skills, whereas 22.2% reported that they base their decision on their knowhow. The majority of interviewed physicians said that they ask others for help when possible and use strategies that were effective in the past. Finally, half of the sample reported that they change their initial decision when new elements emerge.

**Table 2:** Responses to Questions in the First Part of the Questionnaire According to Specialty

Item – Focus of the question	Category	Specialty			Total sample	*p*
		Emergency	Surgery	Internal Medicine		
Item 1 - Average number of decisions [M (SD)]		40.4 (7.3)	31.0% (7.6)	23 (4.4)	31.5 (28.5)	.147
Item 3 - Decisions with direct responsibility		73.2%	59.4%	75.0%	69.2%	.289
Item 4 - Reversible decisions		54.1%	56.4%	58.5%	56.3%	.628
Item 5 - Decisions related to oneself		51.4%	24.2%	41.1%	38.9%	.041
Item 6 - Decisions that require a lot of time		23.9%	21.1%	25.0%	23.4%	.594
Item 7 - Decisions followed by regret		11.8%	15.8%	11.3%	13.0%	.193
Item 2 - Typical decision (frequency)						.003
	admittance/ discharge	8	2	1	11	
	Therapy	0	5	10	15	
	surgery or invasive tests	3	4	1	8	
	Diagnosis	7	7	6	20	
Item 8a - Context (frequency)						.102
	Urgency	5	8	4	17	
	Routine	4	6	10	20	
	management and organizational problems	3	3	3	9	
	Confusion	6	1	1	8	
Item 8b - First thought (frequency)						.980
	not to cause harm	7	7	7	21	
	focus on patient	4	3	4	11	
	ask oneself questions/ reflect	6	8	7	21	
Item 8c - Feelings (frequency)						.411
	Calm	2	3	6	11	
	Stressed	7	7	6	20	
	concentrated/ totally absorbed	4	6	5	15	
	other	5	2	1	8	
Item 8d - What one does to reach a decision (frequency)						.388
	investigate alternatives/ consultation	7	4	6	17	
	Strategy	7	5	8	20	
	accumulated knowhow	2	6	4	12	
	Instinct	2	3	0	5	
Item 8e - Decide alone/ with help (frequency)						.218
	Alone	6	3	7	16	
	with others	6	8	2	16	
	It depends/if I can	6	7	9	22	
Item 8f - Experiment with solutions (frequency)						.366
	effective in the past	9	13	9	31	
	I experiment	2	0	2	4	
	it depends	1	2	4	7	
	Both	6	3	3	12	
Item 8g - Adhere to decisions made (frequency)						.046
	I adhere to my decisions	7	7	2	16	
	I change decisions in progress	7	6	14	27	
	I change after reflection	3	1	0	4	
	it depends	1	4	2	7	

One-way analysis of variance (ANOVA) with medical specialty as an independent variable was carried out using the closed questions in the first part of the questionnaire (Table 2, Items 1 and 3-7). We detected significant differences among the categories of physicians for Item 5 (*F*(2.53) = 3.79, *p* < .05, η^2^ = .18). Bonferroni’s post-hoc test (*p* < .05) showed that decisions related to oneself were more relevant to ER physicians than to surgeons. Although the differences among specialty groups did not reach statistical significance, it is worth noting that according to these self-reported data, the greatest number of decisions are made in ERs, especially compared to the average number of decisions reported by internists. Direct responsibility for decisions did not differ among the three specialist groups. The data indicated the same trend for ER physicians and internists, who reported a greater number of decisions with direct responsibility than surgeons.

The distributions of the categorized responses to the open questions were analyzed with contingency tables and the relative χ^2^ test (Table 2, Items 2 and 8a-g). There were significant differences in the types of decisions made by the specialists (χ^2^(6; *N* = 54) = 19.66, *p* < .005) based on the item “Describe three typical decisions of your working day.” More specifically, ER physicians reported that they more frequently had to make decisions about patient admission or discharge, whereas internists stated that they made more decisions about therapy.

Responses to item 8g (“Once you have made a decision, do you adhere to that decision or do you change it? On what basis of thought process/observations do you change or not change your decision?”) were differently distributed among the specialty groups (χ^2^(6, *N* = 54) = 12.81; *p* < .05); internists reported that they changed decisions more frequently than did the other two specialty groups.

One-way ANOVA including the level of expertise as an independent variable was carried out using the closed questions in the first part of the questionnaire (Table 3, Items 1 and 3-7). Results suggested that younger physicians make a greater number of decisions than older practitioners (*F*(2.53) = 2.98, *p* < .05, η^2^ = .17). Although the differences among expertise groups did not reach statistical significance, younger physicians tended to make fewer decisions with direct responsibility than members of the other two groups.

**Table 3:** Responses to Questions in the First Part of the Questionnaire, According to Level of Expertise

Item – Focus of the Question	Category	Level of Expertise			*p*
		Low	Medium	High	
Item 1 - Average number of decisions [M (SD)]		38.5 (6.8)	30.3 (6.4)	23.7 (6.1)	.049
Item 3 - Decisions with direct responsibility		62.4%	73.9%	73.9%	.190
Item 4 - Reversible decisions		57.0%	58.6%	53.9%	.546
Item 5 - Decisions related to oneself		42.0%	38.3%	35.5%	.245
Item 6 - Decisions that require a lot of time		23.7%	22.2%	23.9%	.764
Item 7 - Decisions followed by regret		12.3%	11.1%	15.4%	.354
Item 2 - Typical decision (frequency)					.211
	admittance/ discharge	6	4	1	
	therapy	3	4	8	
	surgery or invasive tests	3	1	4	
	diagnosis	10	5	5	
Item 8a - Context (frequency)					.097
	urgency	8	4	5	
	routine	4	5	11	
	management and organizational problems	4	3	2	
	confusion	6	2	0	
Item 8b - First thought (frequency)					.982
	not to cause harm	9	5	7	
	focus on patient	5	3	3	
	ask oneself questions/ reflect	8	5	8	
Item 8c - Feelings (frequency)					.306
	Calm	2	2	7	
	stressed	8	6	6	
	concentrated/ totally absorbed	7	4	4	
	Other	5	2	1	
Item 8d - What one does to reach a decision (frequency)					.278
	investigate alternatives/ consultation	6	8	3	
	strategy	8	3	9	
	accumulated knowhow	5	2	5	
	instinct	3	1	1	
Item 8e - Decide alone/ with help (frequency)					.865
	Alone	7	3	6	
	with others	7	5	4	
	it depends/if I can	8	6	8	
Item 8f - Experiment with solutions (frequency)					.848
	effective in the past	12	9	10	
	I experiment	2	0	2	
	it depends	3	1	3	
	Both	5	4	3	
Item 8g - Adhere to decisions made (frequency)					.150
	I adhere to my decisions	9	3	4	
	I change my decision in progress	10	5	12	
	I change after reflection	2	2	0	
	it depends	1	4	2	

The distributions of the categorized responses to the open questions were analyzed with contingency tables and the relative χ^2^ test (Table 3, Items 2 and 8a-g). No significant differences were detected.

### Part 2 of the Questionnaire: Metacognitive Knowledge About the Characteristics of the “Good Decision-Maker”

Data from the entire sample (Table 4) highlighted the image of the “good decision-maker” as a person with experience and competence (57%). Respondents also thought that a good decision-maker was a person who may feel regret (94.5%); self-criticism was considered an important quality that stimulates metacognition and aids learning (43%). In 90.7% of responses, being a good decision-maker was considered to arise from interactions between innate and learned skills. Respondents reported that physicians can become good decision-makers through experience (47%) and consultation with others (30%) and can help others to be good decision-makers by setting a good example (52%) and promoting metacognition and self-esteem (34%). Decisions made after careful thought were considered to be of higher quality (74%), and political figures and people from the past were given as examples of good decision-makers (35%).

**Table 4:** Responses to Questions in the Second Part of the Questionnaire, According to Specialty.

Item – Focus of the question	Category	Specialty			Total Sample	*p*
		Emergency	Surgery	Internal Medicine		
Item 9 - Traits of a good decision-maker (frequency)						.201
	experience/competence	11	11	9	31	
	intuition/strength of character	4	5	2	11	
	intelligence/ metacognitive skills	2	2	7	11	
Item 10 - A good decision-maker feels regret		88.9%	100.0%	94.5%	94.5%	.250
Item 10bis - Feels regret because (frequency)						.856
	one can make mistakes	4	3	5	12	
	one can learn from one’s mistakes	4	5	4	13	
	self-criticism	7	4	8	19	
Item 11 - The best decisions require careful thought		66.6%	66.6%	88.9%	74.0%	.160
Item 12 - The ability of being a good decision-maker is (frequency)						.551
	Innate	1	2	0	3	
	Learned	1	0	1	2	
	Both	16	16	17	49	
Item 13 - How one can become a good decision-maker (frequency)						.484
	Experience	7	10	6	23	
	training/teachers	5	3	7	15	
	increasing one’s metacognitive awareness	5	3	3	11	
Item 14 - How one can help others become good decision-makers (frequency)						.790
	giving advice	5	2	4	11	
	developing self-esteem/ metacognition	5	7	5	17	
	setting an example	7	7	7	21	
Item 15 - Examples of a good decision-maker (frequency)						.768
	colleagues/superiors	3	3	3	9	
	family members/friends	3	4	2	9	
	current politicians	2	2	2	6	
	historical figures	6	7	2	15	
	fantasy/mythological figures	1	0	2	3	

One-way ANOVA was conducted for closed questions (Table 4, Items 9 and 10bis), and the distributions of categorized responses to the open questions were analyzed with contingency tables and the relative χ^2^ test (Table 4, Items 9, 10bis, and 12-15). These analyses uncovered no significance differences. However, ER physicians and surgeons tended to value experience as the major characteristic of a good decision-maker, whereas internists placed greater importance on intelligence and metacognitive skills. Additionally, the entire group of surgeons (100%) considered regret to be a fundamental characteristic of a good decision-maker; fewer ER physicians (88.9%) expressed this opinion.

One-way ANOVA was conducted for closed questions (Table 5, Items 9 and 10bis) to explore the perceived effect of expertise on decision making. The differences among expertise groups were not significant for either item. Our data indicated that all physicians with the highest level of expertise considered the ability to feel regret as fundamental for being a good decision-maker, whereas 90.0% of physicians with low and medium levels of expertise valued regret as a feature of the good decision-maker. Fewer respondents with a medium level of expertise believed that the best decisions require careful thought (64.3%) versus physicians with a high level of expertise (83.3%).

**Table 5:** Responses to Questions in the Second Part of the Questionnaire, Grouped by Level of Expertise.

Item – Focus of the question	Category	Level of Expertise			*p*
		Low	Medium	High	
Item 9 - Traits of a good decision-maker (frequency)					.090
	experience/competence	13	7	11	
	intuition/strength of character	6	3	2	
	intelligence/ metacognitive skills	3	3	5	
Item 10 - A good decision-maker feels regret		90.9%	92.8%	100.0%	.379
Item 10bis - Feels regret because (frequency)					.570
	one can make mistakes	4	3	5	
	one can learn from one’s mistakes	8	1	4	
	self-criticism	8	5	6	
Item 11 - The best decisions require careful thought		72.6%	64.3%	83.3%	.150
Item 12 - The ability of being a good decision-maker is (frequency)					.702
	Innate	2	0	1	
	Learned	1	0	1	
	Both	19	14	16	
Item 13 - How one can become a good decision-maker (frequency)					.554
	Experience	9	7	7	
	training/teachers	9	3	3	
	increasing one’s metacognitive awareness	3	4	4	
Item 14 - How one can help others become good decision-makers (frequency)					.080
	giving advice	7	1	3	
	developing self-esteem/ metacognition	9	5	3	
	being of example	6	5	10	
Item 15 - Examples of a good decision-maker (frequency)					.908
	colleagues/superiors	4	2	3	
	family members/friends	4	1	4	
	current politicians	2	2	2	
	historical figures	5	5	5	
	fantasy/mythological figures	1	0	2	

χ^2^ values were calculated for the distributions of the categorized responses for each open question in terms of expertise (Table 5, Items 9, 10bis, and 12-15). Two non-significant trends emerged. Regarding the ways in which one can help a person become a good decision-maker, physicians with higher levels of expertise mainly opted for “setting an example,” whereas physicians with low levels of expertise preferred reflection and support over self-esteem (*p* = .08). Last, physicians with high and low levels of expertise more often attributed experience and competence to being a good decision-maker as compared to physicians with medium level of expertise (*p* = .09).

## Discussion and Conclusions

The results of the current study highlight several interesting aspects of the metacognition of decision making by physicians. For example, the types of decisions made in the various medical departments were different. Whereas ER physicians reported that they more often make decisions about patient discharge and admission, internists reported that they are more involved with decisions related to therapy. This result is plausible because ER physicians must deal with a large number of acute patients, whereas internists are required to identify a therapy after diagnosis.

The present study underscores that physicians in the ER generally make decisions about themselves more often than internists and surgeons do. This result may be better understood in light of the peculiar characteristics of ERs, where physicians are more often called upon to reflect on and continuously review their conduct (Antonietti, Andolfi, & Colombo, 2014). A further difference between the ER and internal medicine lies in the number of decisions that are changed over time. The predominance of strategic changes following the availability of new data in internal medicine appears understandable due to the more routine structure in which internists operate, which consequently gives them more time to review their position.

Participants reported that most decisions are generally made quickly; few are followed by regret. In all specialties, nearly half of respondents in the present investigation said that the possibility of feeling regret is a significant characteristic of a good decision-maker, likely motivated by the conviction that a careful critical analysis of the decision can lead to an improvement of one’s metacognition skills and therefore in the quality of one’s decisions (Riva, Monti, Iannello, & Antonietti, 2012). The impossibility of concealing surgical errors and the practitioners’ years of experience may explain why the entire sample of physicians with high levels of expertise deemed fundamental the ability to feel regret (Murphy, Stee, & McEvoy, 2007). In line with the literature on anticipated regret (Zeelenberg, 1999), our respondents indicated that the more difficult a decision (in medical decision making, this difficulty could be due to uncertainty about the risks and outcomes of each option), the more likely it is that individuals consider regret to be an integral part of the decision making process. Results from the present study suggest that physicians not only take regret into account when deciding, but consider the emotional experience of regret as a fundamental feature of a good decision-maker. It is likely that both the anticipation and the post-decisional experience of regret may induce decision-makers to make better choices; regret causes them to think and reflect accurately during each step of the decision making process.

As a form of reflection and a balance of costs/benefits, metacognition seems to be an appropriate approach modality to decision making by physicians. The importance of metacognition is confirmed by our observation that younger physicians in particular consider it fundamental to stimulate self-esteem and professional skills in order to become good decision-makers.

In contrast to the assumptions of normative decision making models in which the decision-maker should rationally analyze all pieces of information available at that moment, this study showed that decisions are often based on acquired knowledge and on strategies that were effective in the past, irrespective of possible mismatches between the current situation and previous ones (Riva, Monti, & Antonietti, 2011). Here, experience accumulated over time seems to play a central role in the decision making process; it was highlighted as one of the most important characteristics that a good decision-maker should have (Riva et al., 2014).

The present work is a pilot study, and our findings require further investigation. The major limitation of this study is the sample size, which is relatively small to support broad generalizations. However, we hope that the present research will contribute to an interesting topic that is not yet well described in the literature. We anticipate that these data will be useful for establishing a tentative instrument for physicians to increase their metacognitive awareness in decision making.

In conclusion, based on these self-reported data, we conclude that physicians are aware that they are acting and operating within a context of uncertainty, with a high risk of error. Overall, the current results indicate a certain sensitivity to the attitude of reflection, which respondents deemed useful and effective for providing support to physicians during the decision making process.

## Appendix: Solomon Questionnaire

### Part a: “You as a decision-maker”

#### a1. Descriptive-behavioral section

1. How many decisions connected with your occupation do you make during a day on average? ………
2. Describe briefly three typical decisions you make in your working day:
  I: ………………………………………………….
  II: ………………………………………………….
  III: ………………………………………………….
3. Think about the decisions you make at work in a day:
  - decisions are you the only and direct person responsible for: how many? .....%
  - decisions you share with others the responsibility and the consequences of: how many? .....%
4. Some decisions could be defined as “reversible” since, once you become aware of their ineffectiveness and inappropriateness, you can modify them, partly or entirely; on the contrary, as for other decisions, which can be defined as “irreversible”, once you take them, you can not change them. Think about the decisions you make at work during a day:
  - how many of them are reversible? .....%
  - how many of them are irreversible? .....%
5. Think about the decisions you make at work:
  - how many of them concern exclusively or mainly yourself? ....%
  - how many of them concern also other people? ....%
6. Thinking about the time you spend in making decisions at work:
  - how many of them take a lot of time to be made? ....%
  - how many of them are made quickly and immediately? ....%

#### a2. Procedural-emotional section

1. Thinking about the decisions you make during your working day:
  - how many times do you regret your decisions? ....%
  - how many times don’t you regret your decisions? ....%
2. Keep on thinking about your working day. Identify a typical situation, or at least a situation that you often experience, in which making a decision is really demanding and difficult.
  8a. Describe the general situation, that is, the context in which you are requested to make this specific decision ………………………………………………………
  8b. Which is your first thought? ………………………………………………………
  8c. How do you feel when you make this kind of decision?………………………………………………………
  8d. What do you do to make this decision? ………………………………………………………
  8e. Do you face the situation by yourself or do you ask others for help/advice? ………………………………………………………
  8f. Do you basically employ solutions that turned out to be effective in the past, or do you tend to try out new solutions? ………………………………………………………
  8g. Once you have made the decision, do you follow it, or do you modify it (entirely or partly)? On the basis of which thoughts/reflections do you modify /don’t modify your decision? ………………………………………………………

### Part b: “The good decision-maker in general”

1. In your opinion, which peculiarities characterize those people who are effective in taking their decisions?……………………………………………………………………
2. A good decision maker is someone who never regret his/her decision?
  _ Yes
  _ No
  Why …………………………………………………………………………………………………………
3. Best decisions are:
  _ intuitive
  _ analytical
4. According to your opinion, the competence of being “a good decision maker” is:
  _ innate
  _ learned
  _ partly innate, partly learned
5. If you believe that the competence of making good decisions can be learned or improved, how do you think a person can become a good decision maker? ………………………………………………………
6. How can you help someone to make good decisions? ………………………………………………………
7. Which could be a proper example of a “good decision maker”? (you can mention historical or mythological characters, well-known people, but also colleagues, relatives or friends)………………………………………………………
